# Cutaneous Eruptions as a Consequence of Myeloid Leukemic Infiltration: Differential Diagnosis With a Benign Dermatosis

**DOI:** 10.7759/cureus.100672

**Published:** 2026-01-03

**Authors:** Tiago S Duarte, Cristiana L Matos, Ricardo C Martins

**Affiliations:** 1 Family Medicine, Unidade Local de Saúde de Entre Douro e Vouga (ULSEDV) - Unidade de Saúde Familiar (USF) Alpha, Ovar, PRT

**Keywords:** acute myeloid leukemia, blastic phase, dermatitis, essential thrombocythemia, leukemic infiltration

## Abstract

Leukemia cutis is an uncommon but clinically significant manifestation of hematologic malignancies caused by infiltration of the skin by malignant leukocytes. Its presentation is highly variable and may closely resemble benign inflammatory dermatoses, frequently leading to diagnostic delay.

We report the case of a 76-year-old man with JAK2-positive essential thrombocythemia (ET) who developed persistent, intensely pruritic maculopapular skin lesions initially diagnosed as dermatitis. Despite symptomatic treatment, the lesions progressed and were followed by systemic symptoms. Further investigation revealed acute myeloid leukemia (AML) arising from blastic transformation of ET, with probable cutaneous involvement consistent with leukemia cutis.

This case highlights the importance of maintaining a high index of suspicion when evaluating new or treatment-refractory skin lesions in patients with myeloproliferative neoplasms (MPNs), as early recognition may allow timely diagnosis of leukemic transformation and prompt initiation of appropriate therapy.

## Introduction

Leukemia cutis is defined as the infiltration of the skin by malignant leukocytes and represents a rare but clinically relevant manifestation of hematologic malignancies [1,2,3]. It occurs in approximately 2-3% of patients with acute myeloid leukemia (AML), with higher prevalence reported in monocytic and myelomonocytic subtypes [2,4]. The clinical presentation is heterogeneous, ranging from papules and nodules to plaques, maculopapular eruptions, and diffuse erythema, often mimicking benign inflammatory or infectious dermatoses [2,5].

Myeloproliferative neoplasms (MPNs), including essential thrombocythemia (ET), are chronic clonal disorders characterized by dysregulated myeloid proliferation and an inherent risk of leukemic transformation [1,6]. Leukemia cutis may precede, coincide with, or follow systemic leukemic progression and, in some cases, represents the first clinical manifestation of blastic transformation [3,7].

The presence of leukemia cutis is associated with aggressive disease and adverse prognosis, reflecting extramedullary dissemination and advanced clonal evolution [1,5,8]. Because of its nonspecific clinical appearance, diagnosis is frequently delayed. This case illustrates the diagnostic challenges of leukemia cutis presenting as inflammatory dermatitis and underscores the importance of vigilance among primary care physicians and other frontline clinicians caring for patients with underlying hematologic disease [9].

## Case presentation

A 76-year-old man with JAK2 V617F-positive ET presented with new-onset cutaneous lesions. Cytoreductive therapy with acetylsalicylic acid and hydroxyurea had been discontinued in April 2025 due to gastric intolerance, and treatment with anagrelide (0.5 mg twice daily) was initiated, a cytoreductive agent with established long-term safety and efficacy in ET.

In July 2025, the patient presented to an acute primary care consultation with intensely pruritic maculopapular lesions involving the face and neck. He denied fever, weight loss, night sweats, or other systemic symptoms. Given the clinical appearance and absence of systemic manifestations, a presumptive diagnosis of atopic dermatitis was made. In a telephone reassessment three days later, the patient reported partial symptomatic improvement.

**Figure 1:**
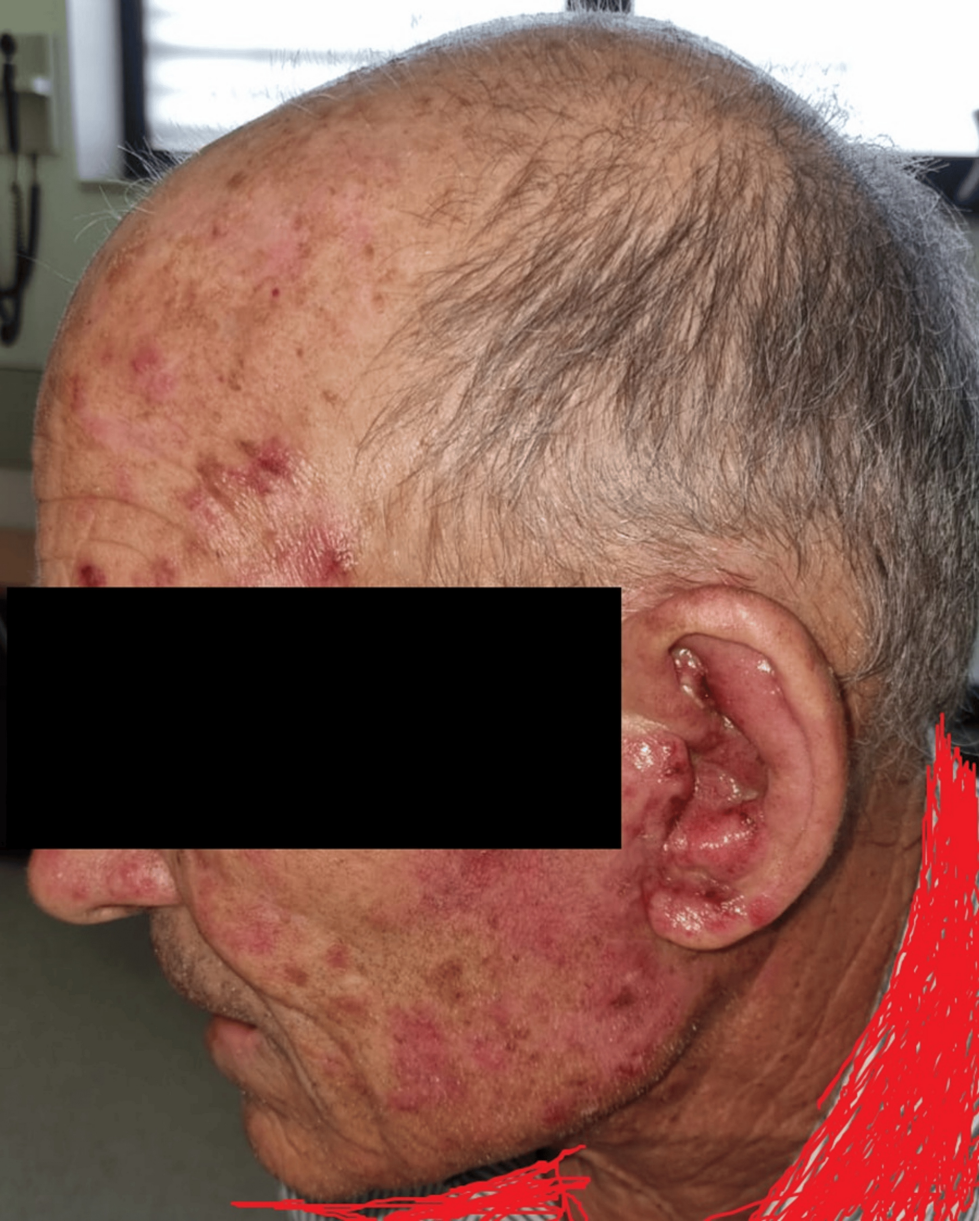
Exuberant inflammatory dermatitis is shown on the face and neck. Note: The patient consented to the use of their images for publication in an open-access journal, and a written, signed consent form was provided to the journal.

**Figure 2:**
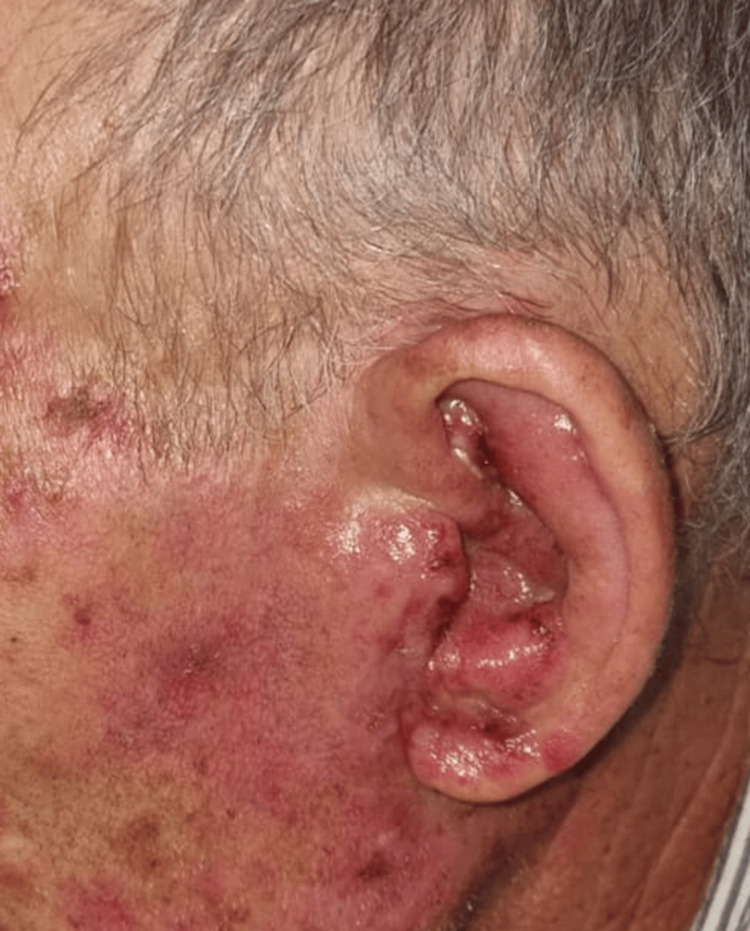
Inflammatory lesions of the left auricle and face Highly inflammatory macular lesions, some with crusting, on the left auricle (external ear), associated with swelling and edema of the same region.

Two weeks later, the patient returned for reassessment due to worsening and extension of the lesions to the trunk (Figures 1, 2). Empirical treatment was broadened to include oral acyclovir and amoxicillin-clavulanic acid to address infectious and drug-related differential diagnoses. A hemogram was also prescribed.

The hemogram revealed anemia, lymphocytosis, monocytosis, and mild thrombocytopenia (Table 1). Suspension of anagrelide was considered but deferred due to the patient already having a scheduled hematology appointment the next week.

**Table 1:** Laboratory results at the second primary care visit A hemogram was ordered by the primary care team. Reference ranges correspond to the laboratory standards.

Laboratory Test	Result	Units	Reference Range
Red blood cells	3.42	×10¹²/L	4.31–6.40
Hemoglobin	10.2	g/dL	13.6–18.0
Hematocrit	30.4	%	39.8–52.0
White blood cells	6.55	×10⁹/L	4.00–10.00
Neutrophils	0.8 (12.1%)	×10⁹/L	1.5–8.0
Lymphocytes	4.2 (64.3%)	×10⁹/L	0.8–4.0
Monocytes	1.5 (23.2%)	×10⁹/L	0.0–1.2
Platelets	132	×10⁹/L	140–440

At the hematology consultation, the patient reported no improvement in skin lesions and had developed systemic symptoms, including persistent fever and oliguria. Given the diagnostic uncertainty of the cutaneous lesions, a structured diagnostic approach to possible leukemia cutis was considered [10]. He was urgently referred to the emergency department, where further investigation revealed AML in blastic transformation. Bone marrow aspiration demonstrated 34.3% myeloid-lineage blasts, consistent with AML arising from ET according to World Health Organization criteria [9]. The cutaneous findings were interpreted as probable leukemic infiltration, consistent with leukemia cutis.

The patient was admitted with febrile neutropenia and treated with broad-spectrum intravenous antibiotics. After clinical stabilization, disease-directed therapy with azacitidine in combination with venetoclax was initiated, leading to the resolution of fever and partial regression of the cutaneous lesions. At the time of writing, the patient remains under close hematology follow-up.

## Discussion

This case illustrates a diagnostically challenging presentation of leukemia cutis occurring in the context of blastic transformation of JAK2-positive ET The initial presentation with intensely pruritic maculopapular lesions closely resembled benign dermatitis, a common condition in elderly patients and a reasonable initial diagnostic assumption.

Leukemia cutis is well recognized for its ability to mimic inflammatory dermatoses such as eczema, urticaria, drug eruptions, and infections [2,5]. This overlap frequently results in delayed diagnosis, particularly when systemic signs of leukemia are absent at presentation [3,6]. Persistent, atypical, or treatment-refractory skin lesions in patients with hematologic disorders should prompt early dermatologic evaluation and consideration of skin biopsy [3,6].

Leukemic transformation of MPNs is associated with cumulative genetic instability, disease duration, and prior cytoreductive therapy [1,8]. Cutaneous infiltration reflects extramedullary dissemination and is often linked to systemic symptoms, cytopenias, and poor prognosis [5,10]. In this patient, progression of skin lesions preceded overt systemic deterioration, highlighting leukemia cutis as a potential early indicator of transformation.

A limitation of this case is the absence of histopathological confirmation of leukemia cutis. Although histopathological confirmation was not available, skin biopsy is considered the gold standard for diagnosis. Typical findings include a dense dermal infiltrate of atypical leukemic cells, most often arranged in a perivascular and periadnexal pattern, with relative sparing of the epidermis. Histologic features may vary according to the leukemia subtype, and immunohistochemistry is commonly required to confirm myeloid lineage and correlate cutaneous findings with systemic disease [2,10]. Nevertheless, the clinical evolution, temporal association with leukemic transformation, and partial regression of skin lesions following systemic therapy strongly support this diagnosis.

Management of leukemia cutis is directed at the underlying hematologic malignancy, as local therapies alone are ineffective [5,10]. Prognosis depends largely on response to systemic treatment, and AML arising from MPNs remains associated with inferior outcomes compared with de novo AML [1].

## Conclusions

Leukemia cutis is a rare but clinically significant manifestation of leukemic transformation that may masquerade as a benign inflammatory skin disease. In patients with MPNs, the appearance of new, persistent, or treatment-resistant cutaneous lesions should raise suspicion for malignant progression.

Early recognition and prompt multidisciplinary evaluation are essential to avoid diagnostic delays and initiate appropriate systemic therapy. This case reinforces the critical role of frontline clinicians in identifying atypical presentations that may represent the first sign of aggressive hematologic disease.
